# Supplementary material for: An Interdisciplinary Assessment of Biochemical and Antioxidant Attributes of Six Greek Vicia sativa L. Varieties
Source: Plants (Basel). 2023 Jul 28;12(15):2807. doi: 10.3390/plants12152807 (PMC10421230; doi:10.3390/plants12152807)
Supplement: Supplementary file 1 [file plants-12-02807-s001.zip › plants-2513094-supplementary.pdf]

Table S1: Results of Bonferroni tests

Total Phenolic Content

Biomass

Anova: Single Factor

SUMMARY

Groups	Count	Sum	Average	Variance
Leonidas	3	0.3996	0.1332	9.48E-06
Line A	3	0.3842	0.128067	0.005661
BI-233	3	0.3645	0.1215	0.000211
Istros	3	0.49	0.163333	6.07E-05
BI-65	3	0.896	0.298667	0.000112
M-6900	3	0.3743	0.124767	1.72E-05

ANOVA

Source of Variation	*SS*	*df*	*MS*	*F*	*P-value*	*F crit*
Between Groups	0.071058	5	0.014212	14.0445	0.000116	3.105875
Within Groups	0.012143	12	0.001012			
Total	0.083201	17				

Since the p-value in the ANOVA table is less than 0.05 (0.000116), we have sufficient evidence to reject the null hypothesis. The mean exam scores between the 6 groups are not equal.

p-values (Bonferroni Test)

	Line A	*BI-233*	*Istros*	*BI-65*	*M-6900*
Leonidas	0.912	0.244	0.003	0.000	0.048
Line A		0.889	0.465	0.018	0.943
BI-233			0.012	0.000	0.727
Istros				0.000	0.002
BI-65					0.000

Grains

Anova: Single Factor

SUMMARY

Groups	Count	Sum	Average	Variance
Istros	3	0.474	0.158	0.000433
BI-65	3	0.3678	0.1226	0.000162
M-6900	3	0.2364	0.0788	0.00115

ANOVA

Source of Variation	*SS*	*df*	*MS*	*F*	*P-value*	*F crit*
Between Groups	0.00944424	2	0.004722	8.119933	0.019636	5.143253
Within Groups	0.00348928	6	0.000582			
Total	0.01293352	8				

Since the p-value in the ANOVA table is less than 0.05 (0.019636), we have sufficient evidence to reject the null hypothesis. The mean exam scores between the 6 groups are not equal.

p-values (Bonferroni Test)

	BI-65	*M-6900*
Istros	0.066	0.026
BI-65		0.104

Total Tannin Content

Biomass

Anova: Single Factor

SUMMARY

Groups	*Count*	*Sum*	*Average*	*Variance*
Line A	3	0.1677	0.0559	4.9E-07
BI-65	3	0.2202	0.0734	3.6E-05
M-6900	3	0.1458	0.0486	4.35E-05

ANOVA

Source of Variation	*SS*	*df*	*MS*	*F*	*P-value*	*F crit*
Between Groups	0.000975	2	0.000487	18.27338	0.002804	5.143253
Within Groups	0.00016	6	2.67E-05			
Total	0.001135	8				

Since the p-value in the ANOVA table is less than 0.05 (0.002804), we have sufficient evidence to reject the null hypothesis. The mean exam scores between the 6 groups are not equal.

p-values (Bonferroni Test)

	BI-65	M-6900
Line A	0.007408	0.129222
BI-65		0.00854

DPPH-Hexane

Biomass

Anova: Single Factor

SUMMARY

Groups	*Count*	*Sum*	*Average*	*Variance*
BI-65	3	3.7	1.233333	9.81E-05
BI-233	3	3.7036	1.234533	0.000295
Istros	3	3.8811	1.2937	0.005685
M-6900	3	3.7702	1.256733	0.002252

ANOVA

Source of Variation	*SS*	*df*	*MS*	*F*	*P-value*	*F crit*
Between Groups	0.007165	3	0.002388	1.146851	0.387541	4.066181
Within Groups	0.01666	8	0.002082			
Total	0.023825	11				

p-values (Bonferroni Test)

	BI-233	*Istros*	*M-6900*
BI-65	0.922	0.241	0.450
BI-233		0.256	0.489
Istros			0.512

DPPH-Methanol

Biomass

Anova: Single Factor

SUMMARY

Groups	*Count*	*Sum*	*Average*	*Variance*
Line A	3	3.343	1.114333	0.000503
BI-65	3	3.2707	1.090233	0.000104
Leonidas	3	3.7456	1.248533	0.000228
Istros	3	3.8103	1.2701	0.000162
M-6900	3	3.1743	1.0581	0.000665

ANOVA

Source of Variation	SS	df	MS	F	P-value	F crit
Between Groups	0.11168	4	0.02792	83.98154	1.18E-07	3.47805
Within Groups	0.003325	10	0.000332			
Total	0.115005	14				

Since the p-value in the ANOVA table is less than 0.05, we have sufficient evidence to reject the null hypothesis. The mean exam scores between the 6 groups are not equal.

p-values (Bonferroni Test)

	BI-65	*Leonidas*	*Istros*	*M-6900*
Line A	0.165	0.001	0.000	0.046
BI-65		0.000	0.000	0.115
Leonidas			0.132	0.000
Istros				0.000

Grains

Anova: Single Factor

SUMMARY

Groups	Count	Sum	Average	Variance
BI-65	3	3.7013	1.233767	6.48E-05
Istros	3	3.739	1.246333	0.000151

ANOVA

Source of Variation	SS	df	MS	F	P-value	F crit
Between Groups	0.000237	1	0.000237	2.191151	0.212911	7.708647
Within Groups	0.000432	4	0.000108			
Total	0.000669	5				

p-value (Bonferroni Test): BI-65, Istros: 0.213

FRAP Hexane

ANOVA

SUMMARY

Groups	Count	Sum	Average	Variance
Leonidas	3	1.2079	0.402633	0.000374
Line A	3	1.2275	0.409167	5.86E-05
BI-233	3	1.4982	0.4994	0.000153

Istros	3	2.9613	0.9871	0.0024
BI-65	3	2.4033	0.8011	0.000206
M-6900	3	2.6366	0.878867	0.000581

ANOVA

Source of Variation	*SS*	*df*	*MS*	*F*	*P-value*	*F crit*
Between Groups	0.989094	5	0.197819	314.5756	2.74E-12	3.105875
Within Groups	0.007546	12	0.000629			
Total	0.99664	17				

Since the p-value in the ANOVA table is less than 0.05, we have sufficient evidence to reject the null hypothesis. The mean exam scores between the 6 groups are not equal.

p-values (Bonferroni Test)

	Line A	*BI-233*	*Istros*	*BI-65*	*M-6900*
Leonidas	0.615	0.002	0.000	0.000	0.000
Line A		0.000	0.000	0.000	0.000
BI-233			0.000	0.000	0.000
Istros				0.003	0.026
BI-65					0.009

Grains

Anova: Single Factor

SUMMARY

Groups	*Count*	*Sum*	*Average*	*Variance*
Istros	3	0.4957	0.165233	3.25E-05
BI-65	3	0.4466	0.148867	1.92E-06
M-6900	3	0.4509	0.1503	3.88E-06

ANOVA

Source of Variation	*SS*	*df*	*MS*	*F*	*P-value*	*F crit*
Between Groups	0.000493	2	0.000246	19.30186	0.002434	5.143253
Within Groups	7.66E-05	6	1.28E-05			
Total	0.00057	8				

Since the p-value in the ANOVA table is less than 0.05, we have sufficient evidence to reject the null hypothesis. The mean exam scores between the 6 groups are not equal.

p-values (Bonferroni Test)

	BI-65	*M-6900*
Istros	0.008	0.013
BI-65		0.361

FRAP Dichloromethane

Biomass

Anova: Single Factor

SUMMARY

Groups	*Count*	*Sum*	*Average*	*Variance*
Leonidas	3	3.7553	1.251767	0.000359
Line A	3	1.13	0.376667	1.09E-05
BI-233	3	1.3259	0.441967	9.54E-06
Istros	3	0.6247	0.208233	7.15E-05
BI-65	3	1.5424	0.514133	0.000149
M-6900	3	0.7942	0.264733	4.87E-05

ANOVA

Source of Variation	*SS*	*df*	*MS*	*F*	*P-value*	*F crit*
Between Groups	2.171577	5	0.434315	4018.958	6.62E-19	3.105875
Within Groups	0.001297	12	0.000108			
Total	2.172874	17				

Since the p-value in the ANOVA table is less than 0.05, we have sufficient evidence to reject the null hypothesis. The mean exam scores between the 6 groups are not equal.

p-values (Bonferroni Test)

	Line A	*BI-233*	*Istros*	*BI-65*	*M-6900*
Leonidas	0.000	0.000	0.000	0.000	0.000
Line A		0.000	0.000	0.000	0.000
BI-233			0.000	0.001	0.000
Istros				0.000	0.001
BI-65					0.000

Grains

Anova: Single Factor

SUMMARY

Groups	Count	Sum	Average	Variance
Istros	3	0.3332	0.111067	9.43E-07
BI-65	3	0.4011	0.1337	2.41E-06
M-6900	3	0.449	0.149667	3.61E-05

ANOVA

Source of Variation	*SS*	*df*	*MS*	*F*	*P-value*	*F crit*
Between Groups	0.002257	2	0.001129	85.89624	3.84E-05	5.143253
Within Groups	7.88E-05	6	1.31E-05			
Total	0.002336	8				

Since the p-value in the ANOVA table is less than 0.05, we have sufficient evidence to reject the null hypothesis. The mean exam scores between the 6 groups are not equal.

p-values (Bonferroni Test)

	BI-65	*M-6900*
Istros	0.000	0.000
BI-65		0.011

FRAP-Methanol

Biomass

Anova: Single Factor

SUMMARY

Groups	Count	Sum	Average	Variance
Leonidas	3	1.6229	0.540967	2.94E-05
Line A	3	2.2685	0.756167	0.001008
BI-233	3	1.079	0.359667	9.43E-07
Istros	3	1.3808	0.460267	4.53E-05
BI-65	3	2.3072	0.769067	0.004026
M-6900	3	2.6313	0.8771	0.005031

ANOVA

Source of Variation	SS	df	MS	F	P-value	F crit
Between Groups	0.618257	5	0.123651	73.15654	1.45E-08	3.105875
Within Groups	0.020283	12	0.00169			
Total	0.638539	17				

Since the p-value in the ANOVA table is less than 0.05, we have sufficient evidence to reject the null hypothesis. The mean exam scores between the 6 groups are not equal.

p-values (Bonferroni Test)

	Line A	*BI-233*	*Istros*	*BI-65*	*M-6900*
Leonidas	0.000	0.000	0.000	0.003	0.001
Line A		0.000	0.000	0.769	0.054
BI-233			0.000	0.000	0.000
Istros				0.001	0.001
BI-65					0.121

Grains

Anova: Single Factor

SUMMARY

Groups	*Count*	*Sum*	*Average*	*Variance*
Istros	3	1.2261	0.4087	1.25E-05
BI-65	3	1.2816	0.4272	0.000428
M-6900	3	0.768	0.256	4.89E-06

ANOVA

Source of Variation	*SS*	*df*	*MS*	*F*	*P-value*	*F crit*
Between Groups	0.052969	2	0.026484	178.2667	4.53E-06	5.143253
Within Groups	0.000891	6	0.000149			
Total	0.05386	8				

Since the p-value in the ANOVA table is less than 0.05, we have sufficient evidence to reject the null hypothesis. The mean exam scores between the 6 groups are not equal.

p-values (Bonferroni Test)

	BI-65	M-6900
Istros	0.202	0.000
BI-65		0.000
